# The New York Institute of Stomatology

**Published:** 1903-12

**Authors:** 

**Affiliations:** The New York Institute of Stomatology


### Reports of Society Meetings.

### THE NEW YORK INSTITUTE OF STOMATOLOGY.

A MEETING of the Institute was held at the Chelsea, No. 222 West Twenty-third Street, New York, on Tuesday evening, June 2, 1903, the President, Dr. J. Morgan Howe, in the chair.

The minutes of the last meeting were read and approved.

Under the head of Communications on Theory and Practice, Dr. C. O. Kimball read two communications from Dr. L. C. Bryan, Basel, Switzerland.

### TREATING SENSITIVE DENTINE.

BY DR. L. C. BRYAN, BASEL, SWITZERLAND.

GENTLEMEN OF THE INSTITUTE OF STOMATOLOGY,—We have used carbolic acid from time immemorial to anæsthetize dentine, but the suggestion of Dr. Jenkins, of Dresden, to use it *hot* has awakened a new interest in this valuable remedy. I have, however, found that applying it hot to a sensitive tooth is painful in itself, and it soon cools, and each time it is reapplied hot it is painful. I therefore use a piece of cotton twice as large as the cavity, and saturating this with carbolic acid, warming it slightly, insert part of the cotton pellet into the sensitive cavity. I now take the Mitchell double-end bulbous copper gutta-percha remover made by Ash & Sons, or a suitable large steel burnisher which will retain the heat. Have another pellet of cotton on the operating-table saturated with carbolic acid. Heat the copper point and apply to the cotton on the table. Carbolic acid volatilizes at a low heat. If the cotton "smokes," the instrument is too hot, and one waits until it ceases to produce a visible vapor. Then it is applied, still hot, to the outer surface of the cotton in the cavity, allowing this to heat up gradually and not to produce pain. The copper point is gradually buried deeper in the carbolic cotton until it is thoroughly heated. When the instrument cools down, repeat the heating, testing, etc., as before, and in two minutes the desired effect will be produced, if *any* effect can be produced on the sensitive dentine. Treatment should be repeated as the excavating proceeds and sensitive surfaces recur.

### LOCATING AND CURING ABSCESS TRACTS.

No doubt many of you have had trouble in deciding on the direction of alveolar abscess or fistula, either to locate the tooth it leads to or the length and depth of an abscess canal. A painless and very satisfactory method I have found to be the following:

Take an ordinary long-pointed gutta-percha pulp-canal point, or make one by cutting a pointed strip from a sheet of base-plate gutta-percha tapering down to a fine point. Dip the whole pin into carbolic acid ninety-five per cent.; do not warm it. Insert the point into the fistulous opening and let the patient push it in himself, press it in, even beyond any point of resistance, down to the butt-end, leaving enough stand out to see the direction in which it extends, and in which direction the canal leads; let it remain thirty seconds; mark the pin at the surface of the gum.

In removing the point, the thin end will be found to have doubled on itself or curled up at the bottom of the canal, and the whole point will be bent in just the curve of the abscess tract and remained curved after removing. Thus one gets the exact depth and direction of the canal from the mark of the gum surface to the doubled-up end of the pin.

The carbolic acid will cauterize the whole tract; the doubled-up loop at the end can now be dipped again into the carbolic acid and reinserted, twisting it around and around until the abscess sack at the end of the root has been cauterized on all its walls and broken up. This will cure most such tracts if the canal of the tooth has been filled. But, if after giving the treatment a trial the abscess does not heal, the same treatment is reapplied to anæsthetize the tract, left for a minute, the point withdrawn, locating by the curve of the pin the exact direction of the tract. Now a small bur in engine can be used to follow up the tract and reach the diseased point and bore away the diseased bone or rough point of apex of root, applying carbolic acid occasionally as one proceeds. The advantage of knowing if the abscess comes from the point or the side of the root or bifurcation of roots or elsewhere is apparent to any one, and this is the only method I know of locating it exactly and measuring the exact depth of the abscess painlessly. It is also useful in fixing the depth of an alveolitis pocket and at the same time anæsthetizing it before operating to remove tartar, which operation is often otherwise an exceedingly painful one. The carbolic acid softens the surface of the pin and allows it to bend to the exact curve of the canal in the bone, and the end to double on itself at the bottom of the tract. The carbolic acid is dissipated quickly by absorption in the tissues, having the pin rigid and springy, so that it retains the curve given to it on insertion when removed.

L. C. Bryan.

Dr. S. E. Davenport would like to make personal acknowledgments to Dr. Bryan for sending these papers. They certainly would be helpful. Dr. Bryan had shown before that he is a man of great originality. While it was possible that some of the gentlemen had used some of these methods fifty years ago, he must confess that the use of gutta-percha points in the abscess tract was entirely new to him. It seemed to be a very valuable idea.

Dr. C. O. Kimball wished, if there were no further remarks to be made upon this subject, to present a little incident of office practice. He had had occasion, many times, to confess faults. This time he wished to confess somebody else's fault. He had recently run across an extremely simple but an extremely serious case of malpractice; nothing but a little tartar left upon the teeth. A gentleman twenty-five years of age, strong, vigorous, and in excellent health, but with a condition of irritation and redness of the gums about some of the teeth, had recently come into his hands for treatment. The patient began immediately to tell of a course of treatment recommended by his former dentist. It seemed that he had noticed this condition of inflammation two years previous, and had called the attention of his dentist, a New York man, by the way, to it. The dentist had made what he termed the pyrozone test, washing the mouth with pyrozone and water, half and half, and white spots had come out upon the gum, whereupon the dentist asserted that the young man was suffering from a disease of the gums and required antiseptic treatment. He recommended the use of a wash of listerine or pyrozone, and that on no account should he brush the gums for fear of injuring them, but that in cleansing the teeth he should use the softest brush obtainable. He stated that the color of the gums indicated this condition; that the patient had been too diligent in cleansing his teeth and had worn away the gums.

Dr. Kimball stated that it seemed a long story for a very little thing, but the case had so startled him that he could not refrain from describing it in detail. It was hard to believe that any man here in New York, who had been in practice six months, could overlook the true cause of such a condition. It was not at all a difficult case. On the anterior surfaces of the upper central incisors was a black line of hard tartar showing at the surface without pushing the gum back. He had simply removed this tartar. Within twenty-four hours the condition of the mouth was entirely different and well on the road towards recovery. Dr. Kimball did not feel that any apology was necessary for bringing this case before the Institute.

Dr. LeRoy mentioned a similar case of so-called malpractice where the tartar around the neck of the teeth was mistaken by the dentist for decay, and the patient was told that these teeth all needed to be filled, and actually did fill some of the teeth so affected. This seemed to him a more serious case of malpractice than the one mentioned by Dr. Kimball.

Dr. George E. Adams, of South Orange, New Jersey, read a paper entitled "Treatment of Deciduous Molars."

(For Dr. Adams's paper, see page 927.)

AL

DISCUSSION.

Dr. E. A. Bogue was pleased to see that Dr. Adams acquired the wisdom of the ages. It was becoming more and more widely known *now,* as it was years ago, that the deciduous teeth are of consequence. This knowledge had gone into decadence like the other lost arts. He was glad it was again thought worthy of consideration, for unless the deciduous teeth remain in health until the time that nature should naturally and healthfully shed them, it is almost impossible to predicate a sound and regular set of permanent teeth.

Dr. C. F. Allan thought the importance of the preservation of the deciduous teeth had been very well presented by the essayist. Dr. Allan wished to emphasize what Dr. Adams had said about the importance of kindly and gentle treatment of little patients. If treated with kindness and tact, they became the most desirable of patients. Indeed, Dr. Allan would prefer, if he were to choose a special line of practice, to work for children.

Dr. Kimball, in connection with this interesting and valuable paper of Dr. Adams's, mentioned the case of a little girl whom he had had in his chair that very day, the daughter of an old patient of his who had been in his hands ever since she herself was a small child. The child had come a year and a half ago, with all her temporary molars very badly decayed. She was in such a nervous state that it was absolutely impossible to do anything with her. She had had trouble with her ears, her eyes, and her nose, so that she had had three different physicians operating on her head. These things had to be done. When she had come to him she was screaming with fright. He felt as Dr. Adams feels, that a little child he could always manage, provided they had not been frightened by some one else. He could do nothing with this child. Finally, he told the mother that a little something, at least, must be done. He instructed her how to apply nitrate of silver to the child's teeth and sent her home. She used nitrate of silver freely, with a little salt afterwards. Last fall he had seen the child, and was then able to get her to let him apply the nitrate of silver himself. She soon learned that a dentist was not such a bad thing after all. To-day he had had no trouble. He was able to cleanse the teeth and get some gutta-percha into one of them. This simply showed two things,—one, that nitrate of silver will check decay and relieve pain in severe and extreme cases; and two, that even the most nervous children, with a little time and patience, can be controlled.

The President called attention to a tooth that illustrated the restraining effect of nitrate of silver upon decayed surfaces. It was a case of remarkable predisposition to decay. The tooth was one that had been treated with nitrate of silver. Decay had begun all around the original cavity, but the surface treated had not been reattacked, but was elevated above the surrounding decayed surface.

Dr. Wheeler stated that, in connection with Dr. Adams's paper, it had occurred to him that exposed pulps in children's teeth could be saved many times where the same was impossible in the case of adults. This could be accomplished by covering the exposure, without pressure, with a mixture of oxide of zinc and oil of cloves or eugenol. He had practised this with success at the clinic at St. Bartholomew's.

Dr. Locherty thought it would be rather hazardous many times to treat a temporary molar, especially with an abscess where necrosis might follow. He mentioned a case that occurred in his work at the clinic, where, upon extraction, he found the anterior buccal root absorbed, while the other roots were practically perfect.

Dr. F. Milton Smith read a paper entitled "Suggestions regarding the Obligations of the Dentist to Himself."

(For Dr. Smith's paper, see page 930.)

DISCUSSION.

Dr. Adams had been very much interested in the paper. Regarding the question of cutting office hours short for recreation, he had frequently promised himself to do so, but found it very difficult. However, he realized that to prolong hours in the office meant to shorten one's life. Regarding the office stool, Dr. Adams spoke very highly, having used one for sixteen years. If he had to discard the stool, he feared his office hours would be very much shortened. Dr. Smith's remarks regarding astigmatism had interested him. For several years he had suffered from severe headaches, so much so that he frequently had to break appointments. His physician said it was indigestion. He had gone to an oculist and had proper glasses fitted. Within a week his headaches had ceased. Before that time scarcely a day ever passed without headache.

Dr. S. E. Davenport expressed his pleasure over the paper, and thought there was too much in it for consideration during what remained of the evening. Dr. Smith had called attention to many little points of importance. Regarding the question of sitting while operating, Dr. Davenport said he owed a debt of gratitude to Dr. Bogue for starting him right on this subject, for he operated in this manner now about one-third of the time. Dr. Davenport thought the chair designed by Dr. Bogue was far preferable to the ordinary stool, affording not only rest for the body but for the feet also, having a foot-rest. This enables the operator, during certain parts of the work, to put his feet up from the floor. Dr. Davenport was glad to hear the subject of petty annoyances brought up. He believed that examinations should never be made except by appointment, unless the dentist had a regular consultation hour, which most dentists could not afford. The patient should understand that the time of the operator is valuable, no matter whether he be making an examination or performing the work. Under, practically, no circumstances should the dentist leave the patient being operated upon, especially if the rubber dam is in position. The patient who comes in might be in a plight, but Dr. Davenport could not imagine a much worse plight than the patient with the rubber dam forced down between the second and third molars, for instance. The dentist's duty was to the sufferer in the chair rather than to the patient who happens in. An opportunity can nearly always be made later on in the day for the relief of suffering. Greater progress is made by attention to the work in hand rather than in the effort to please all who may come in without appointment.

Dr. Bogue thought that the quicker dentists learned that they were professional men, and that their time was valuable, the better it would be for them. Until they felt it they could not expect their patients to feel it. In regard to putting in three or four gold fillings when one would be better, Dr. Bogue recalled the indignation expressed by a gentleman when told that it would cost fifty dollars to fill a central incisor that had lost the filling inserted by Dr. Dwinelle. On examining the chart, it was found that he had inserted three fillings on the cutting edge and two on the sides, making in all five fillings, doing the same work that Dr. Bogue proposed to do with one, but the patient had been perfectly willing to pay this price for the five fillings. Here was a splendid operator who had set us this bad example. Dr. Smith is trying to convince his patients that he is doing honest professional services for them. He does not need to try. The thing would show for itself.

Dr. Brush, under the criticism of putting in several fillings where one would do fitter service, mentioned a case that had come under his observation. One of the items on a bill to which exceptions had been taken was for fourteen fillings in a single molar tooth.

Dr. F. Milton Smith appreciated the kindly words that had been spoken, but since no exceptions had been taken, as a paper calculated to provoke discussion he supposed it was practically a failure.

Dr. Smith was indebted to Dr. Davenport for suggestions relating to the operating-chair. Referring to the matter of putting in five or six fillings in place of one, Dr. Smith had referred to those cases only where a single filling would be much better.

Adjourned.

FRED. L. BOGUE, M.D., D.D.S.,
*Editor The New York Institute of Stomatology.*

